# Understanding risk factors and microbial trends implicated in the development of Whipple-related surgical-site infections

**DOI:** 10.1017/ash.2022.377

**Published:** 2023-03-01

**Authors:** Wendy Feng, Ahmer Irfan, Molly Fleece, Vikas Dudeja, Sushanth Reddy, Salila Hashmi, J. Bart Rose, Rachael A. Lee

**Affiliations:** 1 University of Alabama at Birmingham Heersink School of Medicine, Birmingham, Alabama, United States; 2 Division of Surgical Oncology, University of Alabama at Birmingham, Birmingham, Alabama, United States; 3 Division of Infectious Diseases, University of Alabama at Birmingham, Birmingham, Alabama, United States

## Abstract

**Objective:**

The purpose of this study is to understand the role of risk factors and postoperative complications seen in patients undergoing Whipple procedures in the development of surgical site infections. Our secondary goal was to evaluate whether microbial patterns differed between preoperative antibiotic classes, offering insight into the effectiveness of current practices while promoting antibiotic stewardship.

**Design:**

We performed a retrospective cohort study comparing patients with and without SSIs.

**Setting:**

This study was conducted at a tertiary-care center in the southeastern United States.

**Participants:**

Patients who underwent a Whipple procedure between 2012 and 2021 were acquired from the National Surgical Quality Improvement Program (NSQIP) database.

**Results:**

Patients with a bleeding disorder reported higher SSI rates (*P* = .04), whereas patients with a biliary stent reported lower surgical site infection (SSI) rates (*P* = .02) Those with postoperative complications had higher SSI rates, including delayed gastric emptying (*P* < .001) and pancreatic fistula (*P* < .001). Patients with longer operative times were 1.002 times more likely to develop SSIs (adjusted odds ratio [aOR], 1.002; 95% confidence interval [CI], 1.001–1.004; *P* = .006) whereas surgical indications for malignancy correlated with decreased SSIs risk (aOR, 0.578; 95% CI, 0.386–866) when adjusting for body mass index, surgical indication, and duration of surgical procedure.

**Conclusions:**

Optimizing preoperative management of modifiable risk factors for patients undergoing pancreatoduodenectomies and decreasing operative times may reduce SSI rates and patient and hospital burden. Further research is needed to understand whether stent placement reduces SSI risk in pancreatoduodenectomy.

The Whipple procedure, or pancreatoduodenectomy, is the only curative treatment option for patients with proximal pancreatic, biliary, or ampullary malignancies.^1–3^ Although outcomes for patients undergoing pancreatoduodenectomy have significantly improved with the advancement of surgical techniques, pancreatoduodenectomy remains a high-risk procedure that carries an overall 30%–60% morbidity risk.^4,5^ These percentages are influenced by postoperative complications—most commonly, delayed gastric emptying, pancreatic leak, and surgical site infection (SSI)—which develop in almost half of patients.^1,3,4,6^ Delayed gastric emptying and pancreatic leak development can be predicted, but risk factors are largely nonmodifiable and interrelated. However, the ease and widespread availability of microbial testing offers an opportunity to study SSIs and re-evaluate our ability to prevent and manage infections following pancreatoduodenectomies.

Incidence of SSI is estimated to be 1%–3% of all surgical interventions but tends to be higher in abdominal surgeries.^7,8^ Gastroduodenal procedures confer a unique infection risk and require special consideration when choosing antibiotic prophylaxis due to potential contamination of bowel contents onto a sterile field. This risk is amplified by patient factors such as achlorhydria, bowel perforation, morbid obesity, bleeding, and cancer.^9^ Concerns for postoperative SSIs in pancreatoduodenectomies arise from manipulation of the bile duct more specifically.^10^ It is unsurprising that cultured microbials are overwhelmingly gram-negative bacteria, followed by gram-positive cocci such as *Staphylococcus* spp, *Streptococcus* spp, and *Enterococcus* spp.^7,11^

First- and second-generation cephalosporins have been conventionally used as the perioperative antibiotic choice for pancreatoduodenectomy. Prior studies documented statistically significant differences between experimental and placebo groups, particularly in patients with biliary stents.^11–13^ However, increasing awareness and antibiotic stewardship has prompted concerns over rising local resistance patterns.^14^ In this retrospective study, we sought to understand SSI-related pathogens and their respective antibiotic susceptibilities within our patient population and geographical region. Outcomes of this analysis may serve as a framework for optimizing patients undergoing pancreatoduodenectomies and thus improve patient morbidity while reducing hospital burden.

## Methods

### Data collection

Aggregate data on patients who underwent open pancreatoduodenectomy from a single-institution was obtained from the National Surgical Quality and Improvement Program (NSQIP) database (provided by the American College of Surgeons) over a 10-year period (January 1, 2012, to December 31, 2021). Furthermore, 10 attending surgeons performed all pancreatoduodenectomies.

SSIs were defined using the Centers for Disease Control and Prevention (CDC) guidelines.^13^ The data set specified patients who developed SSIs within 30 days of surgery. SSIs were defined as any superficial or deep wound infection, organ-space infection, or positive microbiological data from drain cultures. Microbial speciation and respective sensitivities were acquired through chart review (Supplementary Fig. 1).

**Fig. 1.**
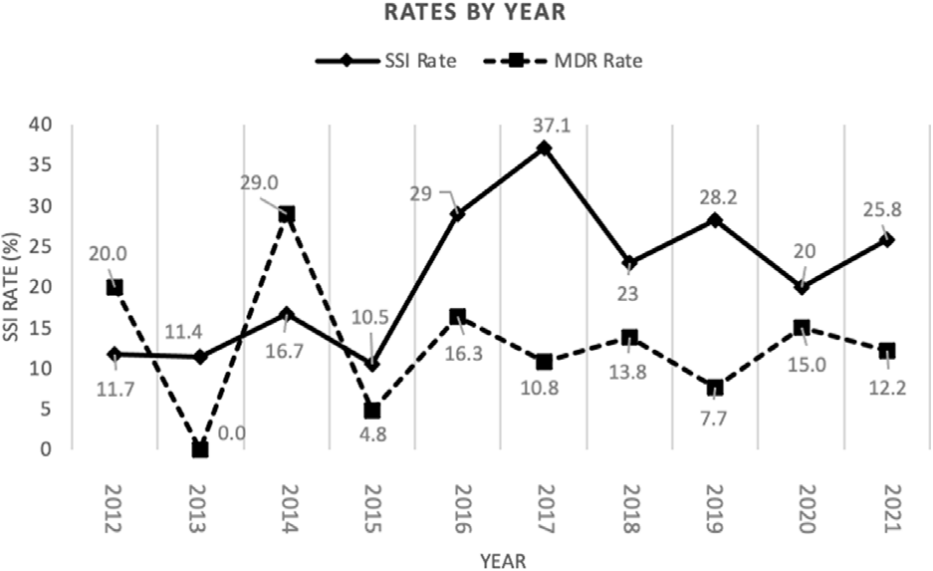
Surgical-site infection (SSI) and multidrug resistance (MDR) rates by year. Superimposed depiction of SSI and MDR rates over 2012–2021. Values represent percentages.

### Statistical analysis

Surgical indications for pancreatoduodenectomy were grouped into categories: cancer or malignancy, benign mass, neoplasm of unknown behavior, pancreatitis, or other. Preoperative antibiotic class was categorized as: first-generation cephalosporins, second- or third-generation cephalosporins, broad-spectrum agents, and unknown. Records with missing preoperative antibiotic information were excluded from certain analyses (n = 322). Organisms were considered multidrug resistant (MDR) if they recorded nonsusceptibility to antimicrobials in 3 or more classes.^15^ *Candida* species were excluded from MDR analysis. Antibiograms were constructed based on sensitivity and resistance patterns gathered from chart review.

Comparison of categorical variables was performed using χ^2^ analysis or the Fisher exact test where appropriate. Continuous variables were tested for normality and were compared using either the Mann-Whitney *U* test for nonparametric results or Student *t* test for parametric results. *P* values <.05 were considered statistically significant. Risk factors were determined for SSI, and multivariable logistic regression models were performed to calculate adjusted odds ratios (ORs) for development of SSI. All statistical analysis was performed using Stata version 16.0 software (StataCorp, College Station, TX).

## Results

### Demographics

In total, 645 patients underwent pancreatoduodenectomies between 2012 and 2021 (Table 1). The mean age of patients was 63.3 years (SD, 11.4) with a balanced distribution of women (48.8%) and men (51.2%). Patient race was self-identified as white (n = 504, 78.1%), black (n = 121, 18.8%), Asian (n = 8, 1.2%), or other or unknown (n = 12, 1.9%). Pancreatoduodenectomy was most frequently performed for cancer (*n* = 445, 69%) followed by benign mass *(n* = 69, 11%) and other (*n* = 69, 11%). Although SSI rates following operative intervention for benign processes were higher, this difference was not statistically significant (*P* = .08).

**Table 1.** Demographics, Risk Factors, Operative Characteristics, and Postoperative Complications Associated With Surgical-Site Infection (SSI)

Variable	Total population (N = 645), No. (%)^a^	No SSI (n = 514), No. (%)^a^	SSI (n = 131), No. (%)^a^	*P* Value
Age, mean y (SD)	63.3 (11.4)	63.4 (11.5)	63.0 (11.2)	.55
Body mass index, mean (SD)	27.9 (6.5)	27.7 (6.7)	28.7 (6.02)	.11
Sex, male	330 (51)	256 (50)	74 (56)	.17
**Race**				.89
White	504 (78)	400 (78)	104 (79)	
Black	121 (19)	99 (19)	22 (17)	
Asian	8 (1)	6 (1)	2 (2)	
Other/unknown	12 (2)	9 (2)	3 (2)	
**Comorbidities**				
Diabetes	195 (30)	152 (30)	43 (33)	.47
Hypertension	381 (59)	301 (59)	80 (61)	.60
Immunosuppressive therapy	16 (2)	11 (2)	5 (4)	.34
Malnourishment	101 (16)	86 (18)	15 (12)	.15
Bleeding disorder	17 (3)	10 (2)	7 (5)	.04
Smoking within 1 year	137 (21)	111 (22)	26 (20)	.66
Presence of biliary stent	(N = 553) 349 (63)	(n = 436) 286 (66)	(n = 117) 63 (54)	.02
Preoperative obstructive jaundice	(N = 581) 357 (61)	(n = 463) 290 (63)	(n = 118) 67 (57)	.24
**Reason for pancreatoduodenectomy**				.08
Cancer	445 (69)	367 (71)	78 (59)	
Benign mass	69 (11)	51 (10)	18 (14)	
Pancreatitis	36 (6)	27 (5)	9 (7)	
Neoplasm of unspecified behavior	22 (3)	14 (3)	8 (6)	
Other	77 (11)	55 (11)	18 (14)	
**Preoperative antibiotics**	(N = 324)	(n = 233)	(n = 91)	.43
First-generation cephalosporin	127 (39)	94 (40)	33 (36)	
Second or third-generation cephalosporin	23 (7)	14 (6)	9 (10)	
Broad spectrum	174 (54)	125 (54)	49 (54)	
**Preoperative laboratory results, mean (SD)**				
WBC	7.6 (3.3)	7.6 (3.0)	7.8 (4.2)	.36
HCT	37.5 (5.2)	37.3 (5.2)	38.3 (5.2)	.05
Platelet	260.7 (94.5)	261.1 (94.2)	259.1 (96.1)	.83
INR	1.05 (0.19)	1.05 (0.2)	1.05 (0.2)	.96
Creatinine	0.89 (0.40)	0.90 (0.43)	0.88 (0.25)	.77
BUN	14.1 (6.3)	13.8 (6.1)	15.2 (7.0)	.03
Albumin	3.7 (0.57)	3.7 (0.6)	3.9 (0.5)	<.001
T bili	1.8 (2.8)	1.81 (2.8)	1.87 (2.8)	.85
AST/SGOT	47.8 (67.5)	48.9 (68.8)	43.6 (61.8)	.44
Alk phos	188.8 (187.7)	190.9 (185.2)	180.4 (198.3)	.58
**Preoperative treatment**				
Chemotherapy	110 (17)	91 (18)	19 (14)	.38
Radiation therapy	15 (2)	14 (3)	1 (0.8)	.21
Surgery duration, mean minutes (SD)	290.0 (123.2)	283.4 (5.3)	315.8 (11.4)	.007
**Wound classification**	(N = 614)	(N = 491)	(N = 123)	
Clean	1 (0.2)	1 (0.2)	0 (0)	
Clean-contaminated	548 (89)	438 (89)	110 (89)	.07
Contaminated	55 (9.0)	47 (9.6)	8 (6.5)	
Dirty/infected	10 (1.6)	5 (1)	5 (4)	
**Complications**				
Delayed gastric emptying	67 (10.4)	32 (6.2)	35 (26.7)	<.001
Fistula	127 (19.7)	64 (13.2)	63 (50)	<.001
Sepsis	35 (0.5)	9 (1.8)	26 (19.8)	<.001
UTI	8 (1.2)	7 (1.4)	1 (0.8)	.59
Pneumonia	16 (2.5)	8 (1.6)	8 (6.1)	.003
Ventilator requirement >48 hours	19 (2.9)	8 (1.6)	11 (8.4)	<.001
Intubated	29 (4.5)	14 (2.7)	15 (11.5)	.002
MI	6 (0.9)	3 (0.6)	3 (2.3)	.09
CVA	3 (0.5)	2 (0.4)	1 (0.8)	.58
PE	2 (0.3)	1 (0.2)	1 (0.8)	.33
Renal insufficiency	10 (1.6)	5 (1)	5 (3.8)	.03
Dialysis requirement	12 (1.9)	4 (0.8)	8 (6.1)	<.001
	106 (16.4)	81 (15.8)	25 (19.1)	.36
Elevated postoperative amylase, median (IQR)	31.5 (264)	26 (117)	526 (5,663)	<.001
Length of hospital stay, median d (IQR)	(N = 639) 8 (5)	(N = 513) 7 (3)	(N = 126) 12 (13)	<.001
Readmissions within 30 d	105 (16)	56 (10.9)	49 (37.4)	<.001

Note. SD, standard deviation; WBC, white blood cell; HCT, hematocrit; INR, international normalized ratio; BUN, blood urea nitrogen; Tbili, total bilirubin; AST/SGOT, aspartate aminotransferase; Alk phos, alkaline phosphate; UTI, urinary tract infection; MI, myocardial infarction; CVA, cardiovascular accident; PE, pulmonary embolism; IQR, interquartile range.

a No. (%) unless otherwise indicated.

### Culture data

**Fig. 2.**
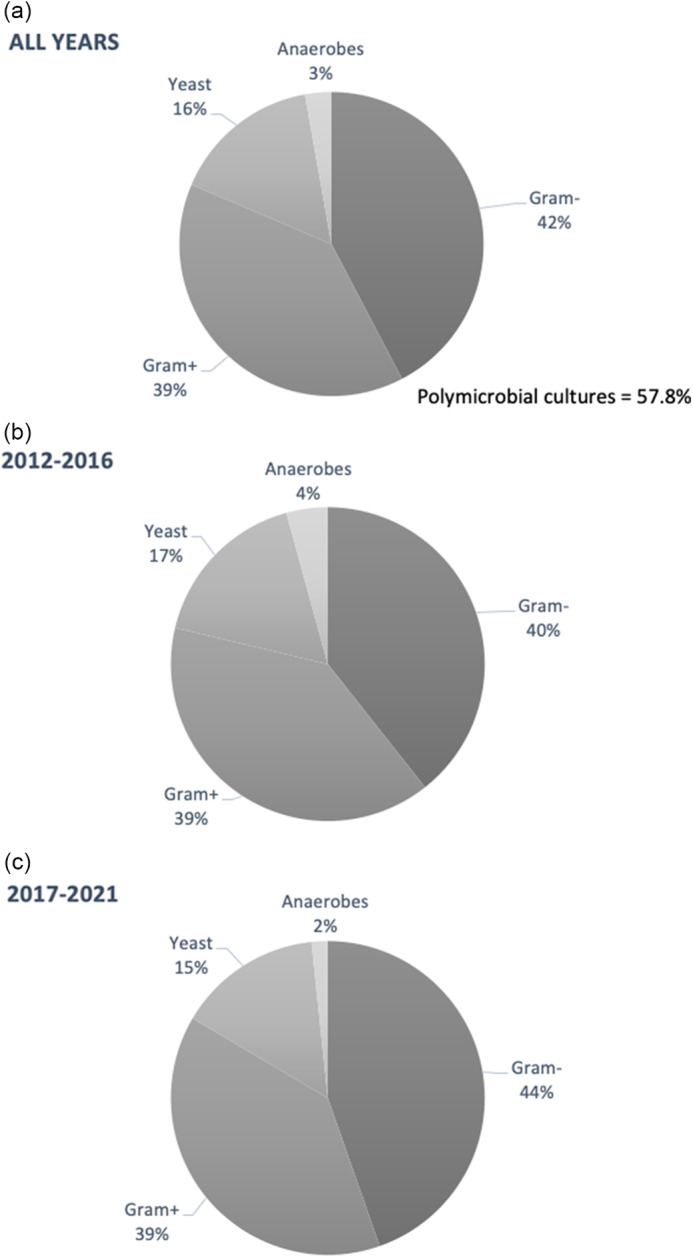
Surgical-site infection (SSI) microbial distributions. Aggregated from (a) 2012–2021 and divided into (b) 2012–2016 and (c) 2017–2021 intervals. Note. gram−, gram negative; gram+, gram positive.

Among 645 patients, 131 (20.3%) developed SSIs, and 75 (57.3%) of those contained culture data documenting speciation and/or antibiotic sensitivities. Of 131 SSI cases, 33 (25%) were considered superficial incisional, 10 (8%) were deep incisional, and 88 (67%) were organ-space infections. Yearly SSI and MDR rates are shown in Figure 1. Highest rates of SSI occurred in years 2016 and 2017 (18.3% and 19.3%; *P* < .001). MDR rates fluctuated drastically between 2012 (20.0%), 2013 (0%), 2014 (29.0%), 2015 (4.8%), and 2016 (16.3%) before stabilizing in 2017 (10.8%), 2018 (13.8%), 2019 (7.7%), 2020 (15.0%), and 2021 (12.2%). Bacteria were most commonly gram-negative isolates (42%) and gram-positive isolates (39%) and were less commonly yeast (16%) or anaerobes (3%) (Fig. 2a). Independent studies from 2012–2016 and 2017–2021 showed similar bacterial distributions (Fig. 2b and 2c). Polymicrobial infections comprised 57.8% of all SSIs. Select patients had their bile ducts cultured intraoperatively at the time of pancreatoduodenectomy. Among those with SSIs, 10 patients had intraoperative bile-duct cultures, of which all returned positive. Only 5 patients were identified to have concordant bacteria causing SSI as bile-duct cultures at the time of pancreatoduodenectomy; all of these patients acquired new pathogens, and 4 of 10 patients acquired MDROs (Table 2).

**Table 2.** Preoperative Biliary Stenting in Patients with Surgical-Site Infection (SSI) (*n* = 10)

Year	Preop Abx Class	Preop Stent Cultures		Postop SSI Cultures		Postop Blood Cultures		Postop Culture Summary		
		No. of Pathogens Cultured	No. of Pathogens Sensitive	No. of Pathogens Cultured	No. of Pathogens Sensitive	No. of Pathogens Cultured	No. of Pathogens Sensitive	No. of Pathogens C/W Preop Cultures	No. of New MDR Pathogens	MDR Pathogen
2012	UTO	5								
2014	UTO	2		6				1/6	1	VRE
2015	UTO	3				1	0/1	2/2		
2017	1st-gen	3	0/3			2	0/2	2/2	1	VRE
2020	BS	3	3/3			0				
2020	1st-gen	2	0/2	1	0/1	0		0/1	1	MRSA
2021	BS	4	3/4	4	3/4			1/4		
2021	1st-gen	5	2/5	3	0/3			2/3	1	*E. coli* (ESBL)
2021	BS	3	2/3	0						
2021	BS	6	5/6	1	0/1			0/1		

Note. MDR, multidrug resistant; UTO, unable to obtain; Abx, antibiotics; Preop, preoperative; Postop, postoperative; 1st-gen, first-generation cephalosporins; BS, broad-spectrum; VRE, vancomycin-resistant *Enterococcus*; MRSA, methicillin-resistant *Staphylococcus aureus*; ESBL, extended-spectrum β-lactamase;

### Preoperative antibiotics

**Fig. 3.**
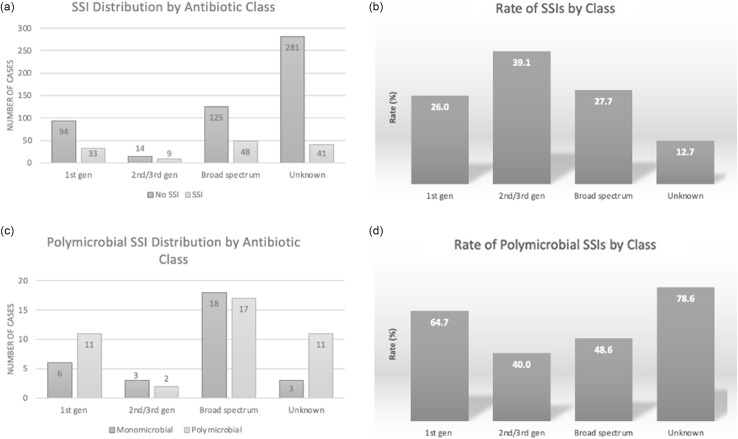
Effect of preoperative antibiotic class. (a) Absolute number and (b) percentages of positive surgical-site infection (SSI) cultures according to preoperative antibiotic class (*P* = .43). Polymicrobial infections incidence is also observed by (c) absolute number and (d) percentages of positive SSIs (*P* = .46).

There were no differences between preoperative antibiotic class and rate of SSIs: first-generation cephalosporins had an SSI rate of 26.0% (uOR, 0.84; 95% CI, 0.51–1.39), second- or third-generation cephalosporins had an SSI rate of 39.1% (uOR, 1.72; 95% CI, 0.72–4.12), and broad-spectrum had an SSI rate of 28.3% (uOR, 1.01; 95% CI, 0.62–1.64; *P* = .43) (Table 1, Fig. 3). Additionally, the rates of polymicrobial infection did not differ between classes (*P* = .46). Microbials cultured from SSIs after administration of the different preoperative antibiotics were variable. When comparing first-generation cephalosporins, second- or third-generation cephalosporins, and broad-spectrum antibiotics, the prevalence differed between *Enterococcus* spp (23% vs 43% vs 18%), *Klebsiella* (18% vs 0% vs 21%), and *Staphylococcus* (3% vs 14% vs 11%). However, the prevalences of *Candida* (12% vs 15% vs 13%) and *Esherichia coli* (14% vs 14% vs 12%) were comparable. Antibiotic sensitivities varied between culture data collected over two 5-year periods (2012–2016 and 2017–2021) as depicted in antibiograms (Supplementary Fig. 2), and a decrease in “resistant” bacteria was demonstrated over time. No significant changes were seen in similar analyses for postoperative blood cultures or *Candida* infections.

### Associations between risk factors and SSIs

Our analyses showed no differences regarding patient demographics or comorbidities among patients with SSI compared to those without SSI, apart from presence of a bleeding disorder (*P* = .04) (Table 1). Patients with preoperative biliary stents had a lower SSI rate (63% vs 57%; uOR, 0.61; 95% CI; 0.40–0.93; *P* = .02;).

Certain preoperative laboratory values were decreased in patients with an SSI, including BUN (*P* = .03), albumin (*P* < .001), and hematocrit (*P* = .05) (Table 1). However, no differences between white blood cell count (*P* = .36), creatinine (*P* = .77), total bilirubin (*P* = .85), or alkaline phosphate (*P* = .58) levels were demonstrated. Preoperative interventions, such as chemotherapy (uOR, 0.79; 95% CI, 0.46–1.3; *P* = .38) or radiation therapy (uOR, 0.27; 95% CI, 0.035–2.08; *P* = .21), were not significant. In our study, the overwhelming majority (89%) of operative fields were considered clean contaminated. No differences in SSI rates were seen between wound classifications: clean, clean-contaminated, contaminated, or dirty-infected (*P* = .07).

In a multivariable analysis adjusting for BMI, surgical indication, and duration of surgical procedure, increased BMI was not predictive of SSI risk (aOR, 1.017; 95% CI, 0.989–1.047; *P* = .24,) (Table 3). A one-minute increase in operative time lead to a 0.2% increase in the odds of developing an SSI (aOR, 1.002; 95% CI, 1.001–1.004; *P* = .006), whereas patients with cancer as a surgical indication had a 42% decrease in the odds of developing an SSI (aOR, 0.578; 95% CI, 0.386–866; *P* = .008).

**Table 3.** Multivariable Logistic Analysis of Predictors for Post-Whipple Surgical-Site Infections (SSIs)

Predictors	Odds Ratio	Confidence Interval	*P* Value
Duration of surgical procedure	1.002	1.001–1.004	.006
Body mass index	1.017	0.989–1.047	.243
Surgical indication	0.578	0.386–0.866	.008

Patients with certain reported postoperative complications also developed SSIs. These included postoperative sepsis (uOR, 13.9; 95% CI, 6.3–30.5; *P* < .001,), pneumonia (uOR, 4.1; 95% CI,1.5–11.2; *P* = .003), ventilator requirement for >48 hours (uOR, 5.8; 95% CI, 2.3–14.7; *P* < .001), intubation requirement (uOR, 2.6; 95% CI, 1.4–4.8; *P* = .002), renal insufficiency (uOR, 4.0; 95% CI, 1.2–14.2; *P* = .03), and dialysis requirement (uOR, 8.3; 95% CI, 2.5–28.0; *P* < .001) (Table 1). Patients undergoing pancreatoduodenectomies are at increased risk for developing complications, and those reporting delayed gastric emptying also had higher rates of SSI (uOR, 5.2; 95% CI, 3.1–8.9; *P* < .001), as did those with fistula formation (uOR, 6.6; 95% CI, 4.2–10.2; *P* < .001). Peak amylase levels measured on postoperative days 2 and 3 were on average statistically different between those with and without SSIs (8,508.5 U/L vs 1,529.5 U/L; *P* < .001). Average length of stay increased by 7.6 days in patients with reported SSIs compared to those without (8.9 vs 16.5 days; *P* < .001). Readmission rate—defined as readmission within 30 days of hospitalization—also increased in patients with SSIs (*P* < .001).

## Discussion

In this retrospective study, we assessed risk factors and microbial patterns of SSIs developing after pancreatoduodenectomies at a single institution over 10 years. From data extracted from the NSQIP database, the overall SSI rate was 20.3%, similar to previously reported rates of 20%–40%.^16^ Stabilized SSI rates in recent years may be related to increased availability and standardization of microbial testing. Moreover, the hospital system in this study transitioned to a new electronic medical record system in 2011, so variability in provider SSI reporting and familiarity with EMR documentation over time may have also contributed to perceived increase in SSI rate over time.^17^

At the start of this study in 2012, our hospital pancreatoduodenectomy protocol called for broad-spectrum as the preoperative antibiotic of choice. In late 2016, a change to first-generation cephalosporins was implemented and later switched back to broad-spectrum in early 2018. During this interval, we observed a surge in the SSI rate from 29.0% in 2016 to 37.1% in 2017 and subsequently down to 23% in 2018. Given missing data on preoperative antibiotics, we could not correlate antimicrobial use with SSI rates. Minor discrepancies between percentages can occur because patients may steer away from protocol-assigned preoperative antibiotics due to patient-specific contraindications (ie, allergies or chronic kidney disease stage 4 or 5 for ertapenem).^18^ Despite changes in SSI rate, the incidence of multidrug-resistant organisms during this interval did not change. The antibiogram format allows visualization of changes in resistance patterns over time (2012–2016 vs 2017–2021). We were able to observe decreases in antimicrobial resistance patterns between year ranges. Interestingly*, Klebsiella oxytoca*, *Pseudomonas*, *E. coli*, and *Citrobacter* spp increased in sensitivities.

Unmodifiable risk factors, such as age, sex, and race, were not linked to post-Whipple SSIs in this study. The data set did not explore socioeconomic status, access to healthcare, or degree of health literacy. These social determinants of health may contribute to patient outcomes and offer important information because our healthcare system serves rural communities in the state of Alabama.^19^ Regarding modifiable risk factors, smoking is strongly correlated with delayed wound healing in the literature, and obesity has been associated with infection, specifically of skin and soft tissue.^20,21^ Interestingly, we did not identify associations between increased BMI or smoking within 1 year and the development of SSIs. Additional patient comorbidities have also been cited as SSI risk factors, such as diabetes mellitus, hypertension, immunosuppression, malnourishment, and bleeding disorders.^8^ Only the presence of bleeding disorder demonstrated a higher SSI rate. This finding may be attributed to standardized preoperative management of the other comorbidities studied compared to bleeding disorders. Guidelines recommending target A1C, blood pressure range, or albumin levels have been well established. Previous reports describe similar increased SSI risks in thoracic and orthopedic cases among patients with bleeding disorders; however, no documentation in abdominal surgeries has been cited.^22,23^ No differences in corresponding preoperative platelet, INR, or PTT values were found between SSI and no SSI cohorts. Nonetheless, low BUN, albumin, and hematocrit were associated with increased SSIs. Collectively, these values illustrate a state of malnutrition either from hypermetabolic states associated with malignancy or decreased oral intake. This discrepancy between laboratory and clinical malnutrition may be due to error in documentation or delayed symptom recognition. Furthermore, the documentation of a comorbidity does not indicate whether patient comorbidities were well managed versus poorly managed and symptomatic, which may be better understood through more objective measures such as preoperative laboratory values. For cancer patients who underwent chemotherapy or radiation therapy within 90 days prior to surgery, no increases in SSI rate were established, reassuring providers that patients may proceed with neoadjuvant therapy to improve cancer outcomes without compromising postoperative infection risk.

Overwhelmingly, the most common indication for undergoing pancreatoduodenectomy is malignancy. Our investigation demonstrates surgical indication of cancer as a negative prognostic factor. This finding has not been previously reported and may warrant further study. Patients with pancreatic head or biliary tract masses often present with preoperative biliary obstruction and may require biliary stenting (metal, plastic, or both). Patients with biliary obstruction did exhibit higher SSI rates; unexpectedly, patients with documented preoperative biliary stents had lower SSI rates in an unadjusted analysis. Although it has been recognized that endoscopic stenting harbors an increased risk of infection by exposing the biliary tree to duodenal bacteria, we hypothesize that confounding variables may be present and are worth investigating.^24,25^

Controversy remains over the choice of preoperative antibiotic therapy as the effectiveness of antibiotic classes is debated, and emerging evidence suggests that institution-specific and/or targeted therapy is more effective in lowering SSI risk than any single antibiotic choice.^26–29^ Intrabdominal surgery most commonly uses early-generation cephalosporins and broad-spectrum antibiotics to cover for gram-positives and/or enterococci and coliform bacteria. Moreover, coverage varies by regional resistance patterns, though antibiotic choices may be limited by patient-specific characteristics (ie, drug reactions, renal insufficiency, or liver disease). There is also concern that preoperative antibiotics may select for bacterial growth. Although we did not detect differences between antibiotic class and SSI rate, bacterial culture and speciation demonstrated a notable discrepancy. Specifically, the use of first-, second-, and third-generation cephalosporins later developed SSIs composed primarily of *Enterococcus* spp and *E. coli*. In contrast, the use of broad-spectrum antibiotics led to *Klebsiella* spp–dominated cultures, followed by *Enterococcus* spp. The rate of polymicrobial infections did not differ according to antibiotic class, suggesting low concern for developing resistance to preoperative antibiotics over time.

We evaluated duration of surgery and wound classification as potential risk factors for developing SSI. As expected, surgery duration was predictive of increased SSI risk; therefore, systemic and team-based approaches to reducing operative length may benefit both patient outcomes and hospital efficiency.^30^ The degree of intraoperative wound contamination has also been suggestive of SSIs in prior studies.^31^ Wounds were categorized as clean, clean-contaminated, contaminated, or dirty-infected. Pancreatic surgeries often involve dividing the bile duct and proximal small bowel, therefore considered clean-contaminated.^32^ The vast majority (89%) of our pancreatoduodenectomies reported clean-contaminated wounds with a 20.1% SSI rate, comparable to our overall 20.3% rate. Unsurprisingly, 5 of 5 dirty or infected wounds developed an SSI.

Almost half of patients undergoing Whipple procedures develop postoperative complications, with delayed gastric emptying and pancreatic leak being the most common.^1,3,4,6^ Pancreatic leaks themselves have been linked to both delayed gastric emptying and SSIs and therefore represents an important prognostic factor to study. Biochemical leaks may be approximated by drain amylase levels monitored in the postoperative course and indicate insufficient pancreatojejunostomy anastomosis. As such, leaked bile, containing autodigestive enzymes, that may seep into surrounding tissues and promote necrosis and bacterial colonization. Our study identified an increase in SSI incidence in patients reporting pancreatic leak, consistent with literature documenting pancreatic leak as an independent risk factor.^33^ Those with increased extra-abdominal postoperative complications related to anesthesia/induction (pneumonia, increased ventilator requirement) or kidney function (renal insufficiency, dialysis requirements) had increased SSI rates. Confounding variables contributing to renal insufficiency include microbial infection or adverse effects of antimicrobial treatment itself. No preoperative differences in creatinine were noted; postoperative creatinine values were not documented. The SSI patient cohort was admitted for 1 week longer on average, further increasing patient morbidity risk.

As predicted, SSI cultures primarily grew gram-negative isolates (*E. coli*) and gram-positive isolates (*Enterococcus* spp), commonly known as culprits of hepatobiliary infection.^34^ Although >99% of gastrointestinal flora are anaerobic, we found that anaerobes only comprised 3% of cultured bacteria.^35^ Therefore, anaerobes are either sufficiently covered by preoperative antibiotics or are difficult to isolate. However, yeasts (specifically *Candida*) make up a considerable percentage of SSIs cultured from our institution, possibly related to antibiotic-induced yeast infections within bowels and superficial skin where they are colonized. More than half of cultures were polymicrobial; polymicrobial infections are common in abdominal surgeries yet do not have inferior outcomes compared to monomicrobial infections.^36–38^ Distributions of bacterial grown from year ranges were comparable, suggesting no significant microbial surge or acquired antibiotic resistance (Fig. 2b and 2c).

This study had several limitations.

Given a patient population of 645, our study utilized patient specific data reported in the NSQIP database. Missing values in this database limited our statistical analyses. Nearly half of our patients (322 of 645) lacked data on preoperative antibiotic class, which restricted our ability to perform logistic regression analyses to evaluable whether choice of preoperative antibiotics was predictive of SSI. Moreover, completion of antibiograms was hindered by unstandardized laboratory testing because bacteria were often tested against different antibacterial agents. For instance, *Pseudomonas* grew from 4 SSI cultures between 2017 and 2021: 2 were resistant to meropenem, and the other 2 were suppressed per laboratory protocol. With data missing from the other 2 cultures, we were unable to calculate a resistance percentage for the antibiogram depiction.

In conclusion,

we identified surgery duration as a prognostic indicator of SSIs in patients undergoing the Whipple procedure. Additionally, we identified a strong correlation between cancer as a surgical indication and decreased SSI rates. This finding has not been reported in previous epidemiologic studies, and future analyses may be warranted. We argue that choice of antimicrobials may be tailored to specific patient needs without compromising SSI risk. Maintaining current Whipple-protocol preoperative antibiotics at our institution while making systematic efforts to decrease operative times may help improve patient outcomes after pancreatoduodenectomy.

## Supplementary material

For supplementary material accompanying this paper visit http://doi.org/10.1017/ash.2022.377. click here to view supplementary material
