# Supplementary material for: Understanding risk factors and microbial trends implicated in the development of Whipple-related surgical-site infections
Source: Antimicrob Steward Healthc Epidemiol. 2023 Mar 1;3(1):e36. doi: 10.1017/ash.2022.377 (PMC10028940; doi:10.1017/ash.2022.377)
Supplement: Supplementary file 1 [file S2732494X22003771sup.zip › S2732494X22003771sup003.docx]

**Supplemental Figures**

**Supplemental Figure 1.**


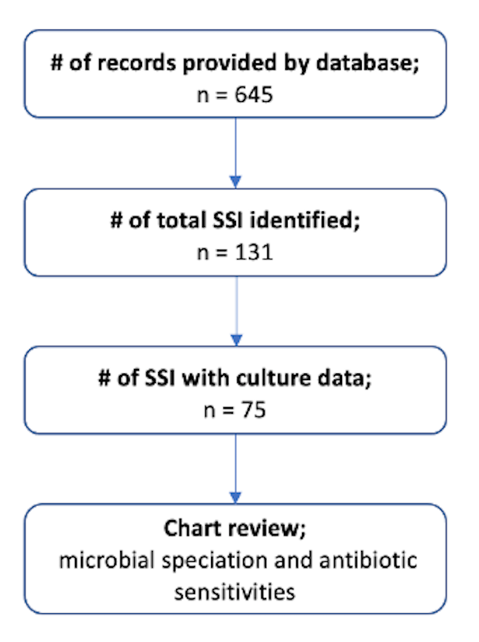


**Supplemental Figure 2.**


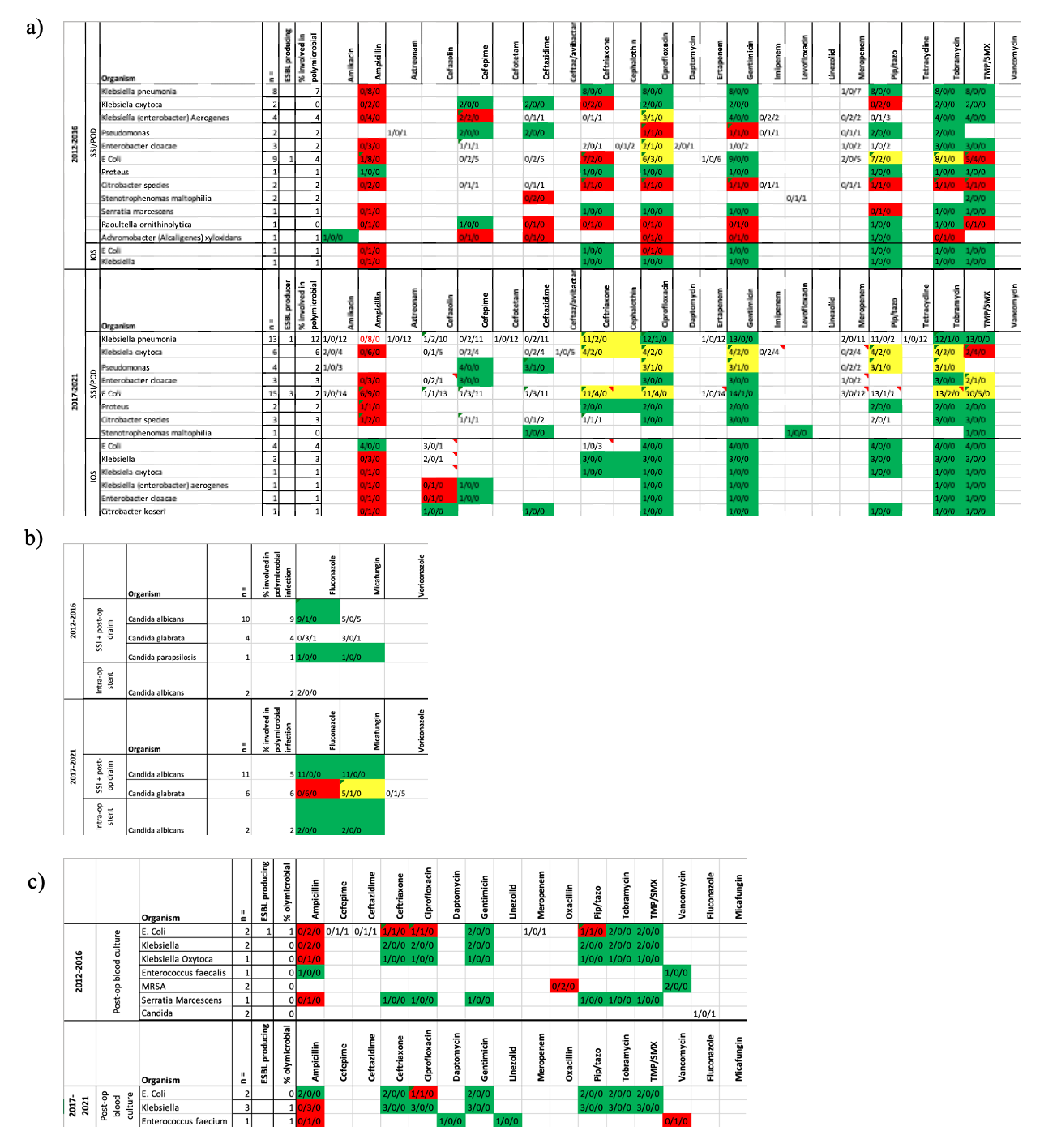


**Supplemental Figure 1.** **Data collection algorithm**.

**Supplemental Figure 2. Antibiogram of cultured gram-negative organisms.** Categorized into (a) SSI gram-negative cultures, (b) candida cultures, and (c) postoperative blood cultures. Culture data is grouped into year ranges 2012-2016 and 2017-2021 and further separated into SSI/postoperative drain culture (SSI and POD) and intraoperative stent (IOS) culture. Percent resistance is depicted by color labeling red ($\leq$60%), yellow (60-90%), red ($\geq$90%). Boxes with missing values remain uncolored. Values are depicted as “susceptible/resistant/unknown.”
