# Supplementary figures and images for: Understanding risk factors and microbial trends implicated in the development of Whipple-related surgical-site infections
Source: Antimicrob Steward Healthc Epidemiol. 2023 Mar 1;3(1):e36. doi: 10.1017/ash.2022.377 (PMC10028940; doi:10.1017/ash.2022.377)

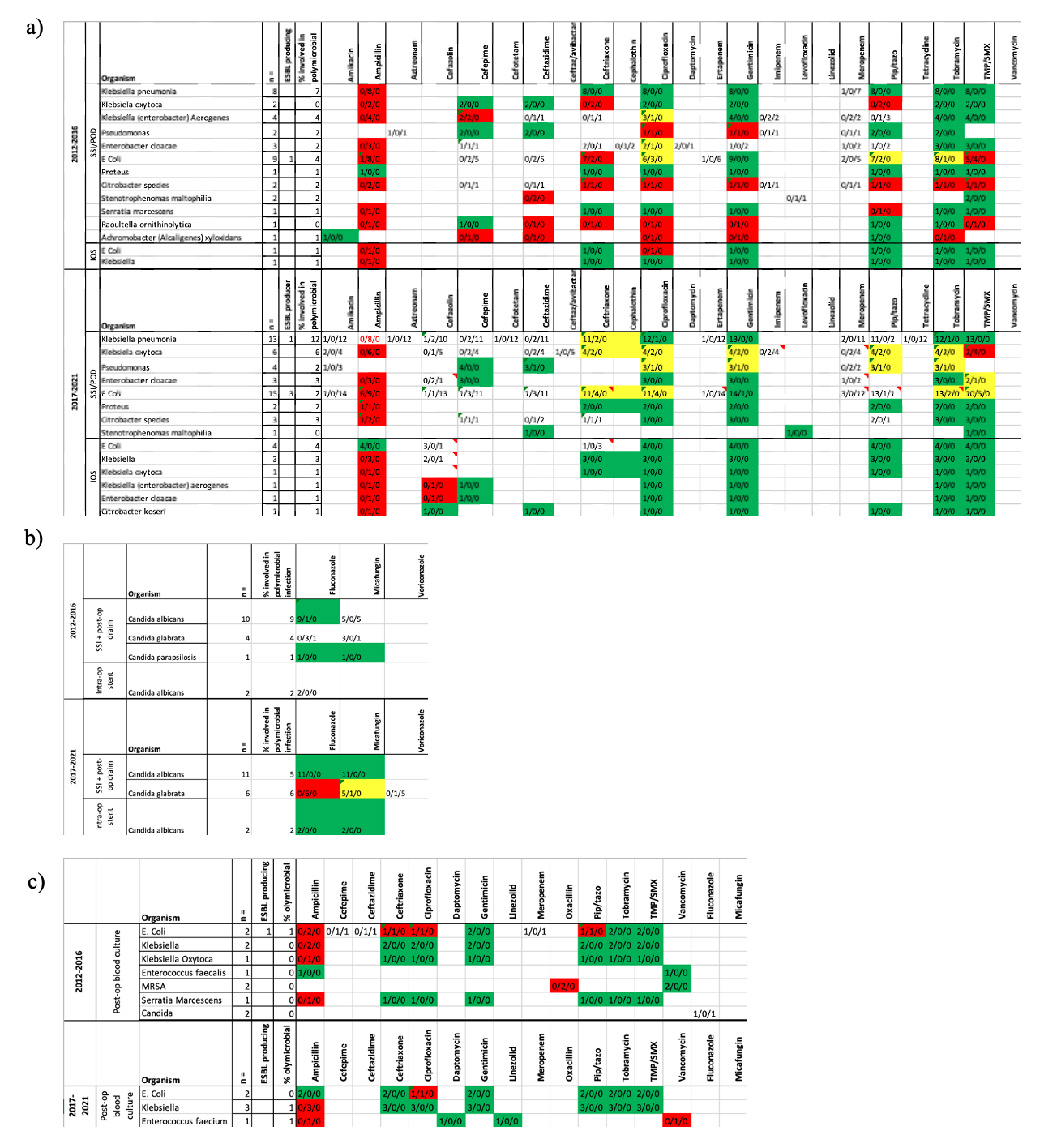

Supplement: Supplementary file 1 [file S2732494X22003771sup.zip › S2732494X22003771sup001.tiff]

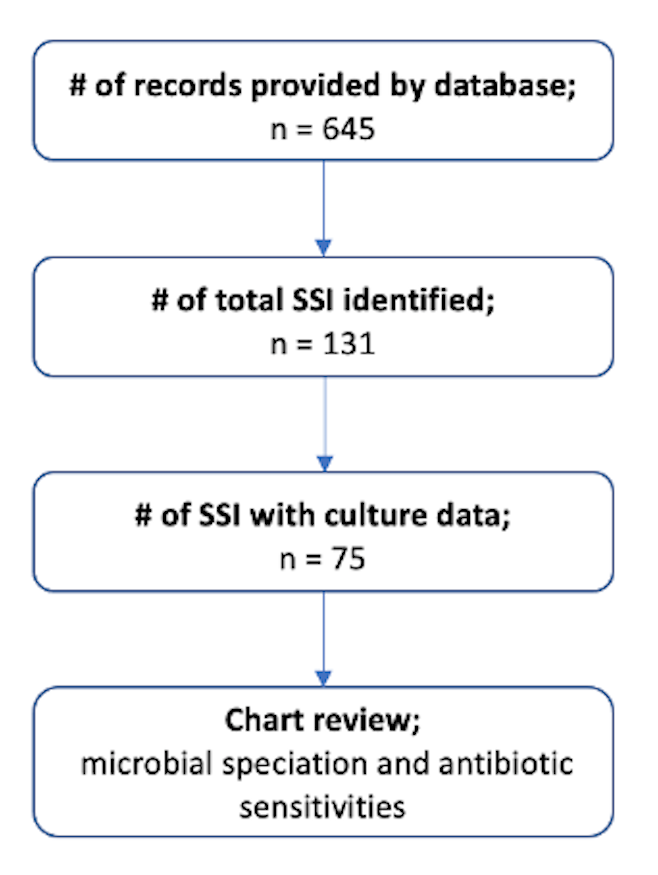

Supplement: Supplementary file 1 [file S2732494X22003771sup.zip › S2732494X22003771sup002.tiff]
